# Comparison of the efficacy of ETS with different segments for palmar, axillary and plantar hyperhidrosis

**DOI:** 10.1186/s12893-023-01976-x

**Published:** 2023-04-11

**Authors:** Ziqiang Hong, Xusheng Wu, Yannan Sheng, Baiqiang Cui, Xiangdou Bai, Yingjie Lu, Tao Cheng, Dacheng Jin, Yunjiu Gou

**Affiliations:** 1 The First Clinical Medical College of Gansu, University of Traditional Chinese Medicine, 35 East Dingxi Road, Lanzhou, Gansu, 730000 China; 2 First Department of Thoracic Surgery, Gansu Provincial Hospital, 204 Donggang West Road, Lanzhou, Gansu, 730000 China

**Keywords:** Endoscopic thoracic sympathectomy, Palmar hyperhidrosis, Axillary hyperhidrosis, Plantar hyperhidrosis, Compensatory hyperhidrosis

## Abstract

**Background:**

To compare the near and long-term outcomes of endoscopic thoracic sympathectomy (ETS) for palmar, axillary and plantar hyperhidrosis.

**Methods:**

We retrospectively analyzed the clinical data of 218 patients with hyperhidrosis who were admitted to the Department of Thoracic Surgery of Gansu Provincial People’s Hospital for surgical treatment from April 2014 to August 2021. The patients were divided into three groups according to the method of ETS and the perioperative clinical data and postoperative follow-up data were collected to compare the near and long term outcomes of the three groups.

**Results:**

There were 197 eligible patients at follow-up, 60 patients in the R4 cut-off group, 95 patients in the R3 + R4 cut-off group and 42 patients in the R4 + R5 cut-off group. There were no statistically significant differences in baseline indicators such as sex, age and positive family history among the three groups (*P* > 0.05). There was no statistically significant difference between the three groups in terms of operative time (*P* = 0.148), intraoperative bleeding (*P* = 0.308) and postoperative hospital stay (*P* = 0.407). Postoperatively, all three groups showed significant relief of palmar hyperhidrosis symptoms, with the R3 + R4 group having an advantage in terms of relief of axillary hyperhidrosis symptoms, patient satisfaction and quality of life index at 6 months postoperatively and the R4 + R5 group having an advantage in terms of relief of plantar hyperhidrosis symptoms. The difference in compensatory hyperhidrosis at 12 months postoperatively was not statistically significant among the three groups (*P* = 0.867), but the incidence was higher in the R3 + R4 and R4 + R5 groups than that in the R4 group.

**Conclusion:**

Patients with simple palmar hyperhidrosis can first consider R4 cut-off treatment; R3 + R4 cut-off is more effective in treating palmar hyperhidrosis combined with axillary hyperhidrosis; R4 + R5 cut-off is more effective in treating palmar hyperhidrosis combined with plantar hyperhidrosis. However, patients need to be informed that R3 + R4 and R4 + R5 dissection may increase the risk of severe compensatory hyperhidrosis after surgery.

## Background

Primary palmar hyperhidrosis is a condition characterized by excessive hyperhidrosis of the palms of the hands. The etiology of primary palmar hyperhidrosis remains unclear. The prevalence of hyperhidrosis in the United States was 2.8% in 2004 [1] and increased to 4.8% in recent years [2]. An epidemiological survey in China showed that the prevalence of palmar hyperhidrosis was 2.08% [3]. Palmar hyperhidrosis often adversely affects the patient’s social interactions, studies and career and may lead to varying degrees of psychological stress.

Patients with palmar hyperhidrosis are often associated with axillary and plantar hyperhidrosis and endoscopic thoracic sympathectomy (ETS) is considered as the most effective treatment for this condition. Different surgical approaches obtain different rates of relief of hyperhidrosis symptoms, with R2-R4 ETS and R3-R4 ETS or ganglion blocks achieving higher rates of relief, but the former can lead to severe postoperative compensatory hyperhidrosis [4, 5]. More research is indispensable to explore the treatment of palmar hyperhidrosis, axillary hyperhidrosis and plantar hyperhidrosis. Our center started ETS in 2014 to treat patients with hyperhidrosis. In this study, we compared the near and long-term efficacy of R4, R3 + R4 and R4 + R5 ETS for palmar hyperhidrosis, axillary hyperhidrosis and plantar hyperhidrosis.

## Patients and methods

### Patients

Clinical data of patients with palmar hyperhidrosis, axillary hyperhidrosis and plantar hyperhidrosis admitted to the Department of Thoracic Surgery of Gansu Provincial People’s Hospital who performed ETS treatment from April 2014 to August 2021 were collected.

Inclusion criteria: (1) according to the clinical guidelines for minimally invasive treatment of palmar hyperhidrosis [6]: patients whose grading criteria are all moderate or severe and who have been treated ineffectively with medication and physical therapy; (2) with varying degrees of axillary and plantar hyperhidrosis, the patient and family have a strong desire for surgery and the indications for surgery are met; (3) complete clinical data (complete surgical records, post-operative follow-up data). Exclusion criteria: (1) intraoperative finding of severe adhesions in the chest cavity; (2) patients with poor cardiopulmonary function or severe cardiac arrhythmias; (3) death due to other causes within the follow-up time.

Diagnostic criteria for palmar hyperhidrosis refer to the clinical guidelines for minimally invasive treatment of palmar hyperhidrosis [6]: (1) bilateral symmetry of hyperhidrosis areas; (2) at least one episode a week; (3) positive family history; (4) cessation of hyperhidrosis during sleep; (5) interference with daily work life, hyperhidrosis of sweat glands visible to the naked eye for more than 6 months without obvious causative factors and meeting the above two manifestations will confirm the diagnosis. We usually diagnose axillary hyperhidrosis and plantar hyperhidrosis according to the patient’s clinical manifestation and clinical data.

**Table 1:** Comparison of clinical data of the three groups of patients [cases(%)/$\bar x$±*s*]

Clinical Information	R4 group (n = 60)	R3 + R4 group (n = 95)	R4 + R5 group (n = 42)	F / *X*^2^ value	*P*-value
Sex				0.874	0.646
male	23 (38.3)	40 (43.1)	20 (47.6)		
female	37 (61.7)	55 (56.9)	22 (52.4)		
Age (years)	22.55 ± 2.74	23.14 ± 3.65	22.69 ± 2.88	0.678	0.509
Age of onset (years)	12.43 ± 1.31	12.30 ± 1.15	12.19 ± 1.35	1.256	0.287
BMI (kg/m^2^)	22.17 ± 2.51	22.45 ± 2.14	22.86 ± 1.97	1.191	0.306
Smoking history	8 (13.3)	10 (10.5)	6 (14.3)	0.282	0.595
Positive family history	7 (11.7)	13 (13.7)	4 (9.5)	0.493	0.782
Symptom severity				0.263	0.877
Serious	32 (53.3)	52 (54.7)	21 (50.0)		
Moderate	28 (46.7)	43 (45.3)	21 (50.0)		
Average follow-up time (months)	14.03	14.33	14.57	1.064	0.347

BMI body mass index.

### Operative methods

All patients underwent thoracoscopic bilateral R4, R3 + R4, or R4 + R5 ETS. Anesthesia was performed by single-lumen tracheal intubation. The patient was placed in a 45-degree semi-Fowler position with the arms abducted 90 degrees to expose the axilla. The surgical incision was chosen as a single incision (third intercostal space in the mid-axillary line). An artificial pneumothorax is created by injecting carbon dioxide into the chest cavity and maintaining the pressure at 10 mm Hg. After identifying the posterior mediastinal sympathetic chain, the mural pleura overlying the sympathetic chain at the 4th rib (R4 group) or the 3rd and 4th ribs (R3 + R4 group) or the 4th and 5th ribs (R4 + R5 group) was hooked and dissected with a conventional 5 mm electric hook and the sympathetic chain was severed by electrocoagulation. The bypassed nerve fibers, such as the Kuntz nerve fiber bundle and sympathetic nerve fibers, are also cut approximately 2–3 cm along the surface of the rib cage. The residual pleural fluid is aspirated with a suction device and the lung is observed under direct vision to reopen the lung without placing a chest drain. The procedure is then repeated on the other side.

### Observation index and efficacy evaluation

(1) Baseline information includes sex, age, age of onset, BMI, smoking history, positive family history, severity of symptoms and follow-up time; (2) the dermatology life quality index (DLQI) [7] was used to assess the improvement of patient’s quality of life after surgery. The DLQI score is designed to score 10 questions based on the impact of the disease on the patient’s life, social and work, with 3 being very severe, 2 standing for severe, 1 representing average and 0 meaning no impact, with scores ranging from 0 to 30, with higher scores suggesting a greater impact of the disease on quality of life [7]. Postoperative compensatory hyperhidrosis is classified into 4 degrees according to the latest clinical guidelines for minimally invasive treatment of palmar hyperhidrosis [6]: mild (grade I): moist skin, no excessive hyperhidrosis or discomfort; moderate (grade II): significant hyperhidrosis and discomfort, but tolerable; severe (grade III): excessive hyperhidrosis, sweat may flow, changing clothes during the day due to excessive hyperhidrosis, but tolerable and not regretting the surgery; very severe (grade IV): excessive hyperhidrosis, sweat may flow, seriously affecting the quality of life, unbearable and regretting the surgery. (3) Prognosis: All patients were followed up for at least 12 months after surgery and follow-up data were collected through questionnaires, physical examinations and outpatient follow-up visits. To compare the relief of hyperhidrosis symptoms, compensatory hyperhidrosis and patient satisfaction in the three groups.

### Statistical analysis

SPSS 26.0 software (SPSS Inc., Chicago, IL, USA) was used for statistical analysis and measurement data conforming to a normal distribution were described by mean ± standard deviation; one-way ANOVA test for comparison between groups and LSD test or Tamhane test for two-way comparison. Categorical variables were expressed as frequencies and percentages (%) and the chi-square test was used for comparison between groups. *P* < 0.05 was considered as a statistically significant difference.

### Ethical review

This study has been reviewed by the Ethics Committee of Gansu Provincial People’s Hospital, approval number: 2022 − 343. All patients signed the informed consent form for surgery before surgery.

**Table 2:** Patient’s surgical outcome and postoperative follow-up results [cases(%)/$\bar x$±*s*]

Observed indicators	R4 group (n = 60)	R3 + R4 group (n = 95)	R4 + R5 group (n = 42)	F value	P value	Two-by-two comparison of P values		
						R4 group vs. R3 + R4 group	R4 group vs. R4 + R5 group	R3 + R4 group vs. R4 + R5 group
Operating time (min)	38.67 ± 5.20	40.05 ± 5.18	40.71 ± 6.68	1.926	0.148	0.131	0.068	0.520
Intraoperative bleeding volume (ml)	7.25 ± 3.25	7.95 ± 3.90	8.33 ± 3.77	1.184	0.308	0.252	0.145	0.572
Postoperative hospital stay (d)	1.28 ± 0.52	1.38 ± 0.57	1.43 ± 0.63	0.902	0.407	0.310	0.206	0.638
Complications	8 (13.3)	14 (14.7)	6 (14.3)	2.447	0.964	0.939	0.910	0.986
Neuralgia	2 (3.3)	4 (4.2)	3 (7.1)					
Pneumothorax	1 (1.7)	2 (2.1)	1 (2.4)					
Pneumonia	5 (8.3)	7 (7.4)	2 (4.8)					
Wound infection	0	1 (1.0)	0					
PAH relief				3.130	0.536	0.420	0.405	0.768
Complete	56 (93.3)	93 (98.0)	41 (97.6)					
Partial	2 (5.0)	1 (1.0)	1 (2.4)					
Unchanged	2 (1.7)	1 (1.0)	0					
AXH relief				1.422	0.840	0.523	0.568	0.760
Complete	17 (28.3)	34 (35.8)	14 (33.3)					
Partial	15 (25.0)	24 (25.3)	12 (28.6)					
Unchanged	28 (46.7)	37 (38.9)	16 (38.1)					
PLH relief				4.764	0.112	0.063	0.008	0.240
Complete	18 (30.0)	40 (42.1)	19 (45.2)					
Partial	14 (23.3)	22 (23.2)	12 (28.6)					
Unchanged	28 (46.7)	33 (34.7)	11 (26.2)					
Patient Satisfaction				4.768	0.092	0.108	0.080	0.132
Satisfaction	54 (90.0)	93 (97.9)	40 (95.2)					
Dissatisfaction	6 (10.0)	2 (2.1)	2 (4.8)					
Preoperative DLQI	16.28 ± 0.94	16.08 ± 1.06	15.95 ± 1.04	1.397	0.250	0.237	0.108	0.486
DLQI at 6 months postoperatively	6.30 ± 0.93	4.77 ± 1.35	4.98 ± 1.37	29.626	< 0.001	< 0.001	< 0.001	0.367
CH(6months postoperative)	45 (75.0)	74 (77.9)	32 (76.2)	0.939	0.988	0.910	0.923	0.990
Mild	17 (28.3)	28 (29.5)	10 (23.8)					
Moderate	25 (41.7)	39 (41.0)	19 (45.2)					
Severe	3 (5.0)	7 (7.4)	3 (7.2)					
CH(12months postoperative)	42 (70.0)	69 (72.6)	30 (71.4)	1.215	0.976	0.912	0.920	0.983
Mild	16 (26.7)	25 (26.3)	10 (23.8)					
Moderate	24 (40.0)	38 (40.0)	18 (42.8)					
Severe	2 (3.3)	6 (6.3)	2 (4.8)					

AXH Axillary hyperhidrosis, CH Compensatory hyperhidrosis, DLQI dermatology life quality index, PAH Palmar hyperhidrosis, PLH Plantar hyperhidrosis.

## Results

### Patient General Information

There were no statistically significant differences in baseline indicators such as sex, age and positive family history among the three groups (*P* > 0.05), see Table 1. Among 68 patients receiving R4 ETS, 60 patients (88.2%) were successfully followed up and 8 patients (11.8%) were lost; Among 106 patients receiving R3 + R4 ETS, 95 patients (89.6%) were successfully followed up and 11 patients (10.4%) were lost; Of the 44 patients receiving R4 + R5 ETS, 42 (95.5%) were successfully followed up and 2 (4.5%) were lost.

### Intraoperative status and postoperative follow-up

All three groups of enrolled patients completed the surgery successfully without perioperative deaths. There was no statistically significant difference between the three groups in terms of operative time (*P* = 0.148), intraoperative bleeding (*P* = 0.308) and postoperative hospital stay (*P* = 0.407). In terms of postoperative complications, the overall incidence was 13.3% in the R4 group, 14.7% in the R3 + R4 group and 14.3% in the R4 + R5 group, with no statistically significant difference among the three groups (*P* = 0.964); see Table 2.

There was no statistically significant difference in the remission of palmar hyperhidrosis in all three groups after surgery (*P* = 0.536); no statistically significant difference in the remission of axillary hyperhidrosis in the three groups (*P* = 0.840), but the rate of complete remission was better in the R3 + R4 group; no statistically significant difference in the remission of plantar hyperhidrosis in the three groups (*P* = 0.112), but the rate of complete remission was better in the R4 + R5 group. Satisfaction was higher in all three groups without statistically significant difference (*P* = 0.092), with the highest satisfaction in the R3 + R4 group. In terms of DLQI at 6 months postoperatively, the difference among the three groups was statistically significant (*P* < 0.001) and the improvement in postoperative quality of life was better in the R3 + R4 group than in the R4 and R4 + R5 groups. All 197 patients in this study has no recurrence after surgery. The overall incidence of compensatory hyperhidrosis at 12 months postoperatively was not statistically significant among the three groups (*P* = 0.976), but the incidence of severe postoperative compensatory hyperhidrosis was higher in the R3 + R4 group (6.3%) and the R4 + R5 group (4.8%) than in the R4 group (3.3%); see Table 2.

## Discussion

Primary hyperhidrosis can affect several parts of the body, such as the palms of the hands, axillae and soles of the feet [8]. Palmar hyperhidrosis is the most common disorders, probably because the hands are the most exposed and commonly used part of the body. As a result, the incidence of hyperhidrosis in other parts of the body is often underestimated, especially in the axillary and plantar regions. Numerous studies have shown that ETS is a safe, effective and minimally invasive method for treating primary hyperhidrosis [9–11].

According to the Chinese clinical guidelines for minimally invasive treatment of palmar hyperhidrosis [6], R3 or R4 ETS was recommended as an effective method for treating palmar hyperhidrosis. The difference is that the palms are drier after R3 and the incidence and severity of compensatory hyperhidrosis is higher than that in R4; a few patients have slightly moist palms after R4, but compensatory hyperhidrosis is less severe than that in R3 resection [6]. The treatment outcome varies depending on the plane of sympathetic nerve cut, so the choice of plane of cut is crucial for patients with surgically treated hyperhidrosis. We noted that hyperhidrosis in the axillary and plantar regions also has a strong negative impact on the mental health of the patients, who has more complaints. However, in the actual treatment process, people often only pay attention to the treatment of hand sweat, but ignore the effect of the treatment of armpit sweat and foot sweat, so the choice of the relevant procedure is also crucial to the effect of the treatment of foot sweat and armpit sweat.

In this study, all three groups of enrolled patients completed the surgery successfully without perioperative deaths. The results of this study showed that there was no statistically significant difference among the three groups in terms of operative time, intraoperative bleeding, postoperative hospital stay and postoperative complications. This indicates similar perioperative outcomes in the three groups. The preoperative DLQI and 6-month postoperative DLQI of the three groups differed significantly, indicating that the postoperative quality of life of the three groups improved significantly, but the postoperative quality of life of the R3 + R4 group improved better than that of the R4 + R5 and R4 groups.

All 197 patients in this study has no recurrence after surgery. Postoperative recurrence is often thought to be related to improper surgical operation of ETS, such as incomplete dissection of the thoracic sympathetic nerve trunk or partial lateral branching, residual nerve regeneration and neuroanatomical variation, suggesting that the operator should try to accurately and adequately dissect the thoracic sympathetic nerve chain on the rib surface and its collateral fibers within 2 cm lateral to avoid thoracic sympathetic nerve regeneration under direct vision during the operation.

A previous study [12] found that R3 + R4 ETS was effective in treating palmar hyperhidrosis, but did not collect data on remission of axillary and plantar hyperhidrosis. Another study [13] included 1,274 patients to explore the option of thoracic sympathectomy. The results of this study showed complete or partial remission of palmar hyperhidrosis symptoms in most patients after R2 + R3 or R3 + R4 dissection and higher remission of plantar hyperhidrosis symptoms in patients who underwent R3 + R4 dissection. The results of this study showed that all three methods significantly improved palmar hyperhidrosis, but R4 ETS alone provided poor relief for axillary and plantar hyperhidrosis. Patients in the R3 + R4 group have higher rate of remission of axillary hyperhidrosis and patients in the R4 + R5 group have higher rate of remission of plantar hyperhidrosis. An anatomical study [14] found that the Kuntz nerve fiber bundle originated from the R3 sympathetic nerve trunk in 10% of patients and that there were many other abnormal pathways between the superior thoracic and intercostal nerves in addition to the Kuntz nerve fiber bundle. The incidence of anatomical variants in the ascending or descending branches of the 2nd, 3rd and 4th ganglia was 54%, 24% and 14%, respectively [15]. Similar findings have been found in other studies [16]. These anatomical variants are clinically important and should be specifically considered when planning an ETS for hyperhidrosis to avoid recurrence of symptoms.

The complete or partial remission of palmar hyperhidrosis, axillary hyperhidrosis and plantar hyperhidrosis after ETS can also be explained by a negative feedback mechanism. As the patient’s postoperative palmar hyperhidrosis subsides, the stress of psychological and social factors is reduced. The improvement in the patient’s emotional and psychological state further contributed to the relief of axillary and plantar hyperhidrosis symptoms. Although ETS treatment improves the symptoms of patients with palmar hyperhidrosis, axillary hyperhidrosis and plantar hyperhidrosis, compensatory hyperhidrosis is a problem that needs to be faced after the procedure. The results of this study showed that 6.3% of patients in the R3 + R4 group, 4.8% of patients in the R4 + R5 group and 3.3% of patients in the R4 group have severe compensatory hyperhidrosis, which is similar to data from previous studies [17]. In addition, the sympathetic trunk is continuously joined by nerve trunks from low to high levels up to the stellate ganglion and the higher level of severance, the greater extent of surgical desympatheticization and the greater compensatory hyperhidrosis after surgery. This could explain the results of our study, with a higher incidence of severe compensatory hyperhidrosis in R3 + R4 ETS and R4 + R5 ETS compared with R4 ETS. The patient’s compensatory hyperhidrosis will also decrease considerably over time and will remain stable for about 6 months after surgery. The incidence of compensatory hyperhidrosis also depends on climatic conditions and emotional stress, with a higher incidence in warm, humid places. We recommend that patients suffering from severe compensatory hyperhidrosis should do the following: (1) avoid excessive physical activity for 1 year after surgery; (2) wear clothes that are breathable and dry quickly; (3) carry a towel with you to wipe sweat. These suggestions are very helpful in relieving the discomfort caused by compensatory hyperhidrosis.

There are certain drawbacks and shortcomings of this study: (1) this study is retrospective, the sample size of the included studies is relatively small and the data source is a single center and the results may be biased; (2) there was no objective assessment of the psychological impact of patient hyperhidrosis before and after surgery; (3) the family history of hyperhidrosis patients in this study is small, which may be related to the patients’ unfamiliarity with hyperhidrosis in family members (mild and some moderate hyperhidrosis without medical consultation and clear diagnosis) during the patient interview; (4) follow-up data were collected through telephone interviews, micro-surveys and outpatient visits. During the follow-up visits, patients usually need to recall their postoperative conditions, which may lead to bias.

## Conclusion

In summary, patients with simple palmar hyperhidrosis can first consider R4 cut-off treatment; R3 + R4 cut-off treatment for palmar hyperhidrosis combined with axillary hyperhidrosis is more effective; R4 + R5 cut-off treatment for palmar hyperhidrosis combined with plantar hyperhidrosis is more effective. However, patients need to be informed that R3 + R4 and R4 + R5 dissection may increase the risk of severe compensatory hyperhidrosis after surgery. Future high-quality prospective randomized controlled trials are expected to further validate the results of this study.

## Data Availability

The datasets used and/or analysed during the current study are available from the corresponding author on reasonable request.

## Abbreviations

AXH: Axillary hyperhidrosis
BMI: body mass index
CH: Compensatory hyperhidrosis
DLQI: dermatology life quality index
ETS: endoscopic thoracic sympathectomy
PAH: Palmar hyperhidrosis
PLH: Plantar hyperhidrosis
